# TRB3 mediates vascular remodeling by activating the MAPK signaling pathway in hypoxic pulmonary hypertension

**DOI:** 10.1186/s12931-021-01908-4

**Published:** 2021-12-14

**Authors:** Xiaopei Cao, Xiaoyu Fang, Mingzhou Guo, Xiaochen Li, Yuanzhou He, Min Xie, Yongjian Xu, Xiansheng Liu

**Affiliations:** 1 Department of Pediatrics, Tongji Hospital, Tongji Medical College, Huazhong University of Science and Technology, Wuhan, China; 2 Department of Pulmonary and Critical Care Medicine, Tongji Hospital, Tongji Medical College, Huazhong University of Science and Technology, Wuhan, China; 3 Key Laboratory of Respiratory Diseases, National Ministry of Health of the People’s Republic of China and National Clinical Research Center for Respiratory Disease, Wuhan, 430030 China

**Keywords:** PH, Hypoxia, PASMCs, TRB3, MAPK

## Abstract

**Background:**

Hypoxic pulmonary hypertension (PH) is a refractory pulmonary vascular remodeling disease, and the efficiency of current PH treatment strategies is unsatisfactory. Tribbles homolog 3 (TRB3), a member of the pseudokinase family, is upregulated in diverse types of cellular stresses and functions as either a pro-proliferative or pro-apoptotic factor depending on the specific microenvironment. The regulatory mechanisms of TRB3 in hypoxic PH are poorly understood.

**Methods:**

We performed studies using TRB3-specific silencing and overexpressing lentiviral vectors to investigate the potential roles of TRB3 on hypoxic pulmonary artery smooth muscle cells (PASMCs). Adeno-associated virus type 1(AVV1) vectors encoding short-hairpin RNAs against rat TRB3 were used to assess the role of TRB3 on hypoxic PH. TRB3 protein expression in PH patients was explored in clinical samples by western blot analysis.

**Results:**

The results of whole-rat genome oligo microarrays showed that the expression of TRB3 and endoplasmic reticulum stress (ERS)-related genes was upregulated in hypoxic PASMCs. TRB3 protein expression was significantly upregulated by hypoxia and thapsigargin. In addition, 4-PBA and 4μ8C, both inhibitors of ERS, decreased the expression of TRB3. TRB3 knockdown promoted apoptosis and damaged the proliferative and migratory abilities of hypoxic PASMCs as well as inhibited activation of the MAPK signaling pathway. TRB3 overexpression stimulated the proliferation and migration of PASMCs but decreased the apoptosis of PASMCs, which was partly reversed by specific inhibitors of ERK, JNK and p38 MAPK. The Co-IP results revealed that TRB3 directly interacts with ERK, JNK, and p38 MAPK. Knockdown of TRB3 in rat lung tissue reduced the right ventricular systolic pressure and decreased pulmonary medial wall thickness in hypoxic PH model rats. Further, the expression of TRB3 in lung tissues was higher in patients with PH compared with those who have normal pulmonary artery pressure.

**Conclusions:**

TRB3 was upregulated in hypoxic PASMCs and was affected by ERS. TRB3 plays a key role in the pathogenesis of hypoxia-induced PH by binding and activating the ERK, JNK, and p38 MAPK pathways. Thus, TRB3 might be a promising target for the treatment of hypoxic PH.

## Introduction

Hypoxic pulmonary hypertension (PH) is a refractory pulmonary vascular remodeling disease that is associated with increased pulmonary artery pressure and pulmonary vascular resistance, ultimately leading to right ventricular failure and premature death [1]. Among the cell types found to participate in the pathological remodeling of pulmonary arteries, pulmonary artery smooth muscle cells (PASMCs) are considered to play a critical role, showing excessive proliferation and migration as well as apoptotic deficiencies [2, 3]. Although progress has been made to understand the precise pathogenesis of PH and to improve the treatment of PH, the efficiency of the current strategies and the prognosis of PH remain unsatisfactory. Therefore, exploring new therapeutic targets and their molecular mechanisms in the pathogenesis of hypoxic PH is particularly urgent.

Hypoxia is a noted stimulus for pulmonary vascular remodeling; however, the cellular and molecular mechanisms involved are still incompletely understood. Hypoxia and other cellular stresses, such as disturbances in calcium homeostasis and oxidative stress, can impair the function of the endoplasmic reticulum (ER) and cause the accumulation of unfolded and/or misfolded proteins, a condition referred to as endoplasmic reticulum stress (ERS) [4]. Our previous study and many other studies have indicated that ERS mediates the pathological process of hypoxic PH [5, 6].

Tribbles homolog 3 (TRB3), a member of the pseudokinase family, is considered to be a sensor of stress induced by hypoxia or ERS [7, 8]. TRB3 is upregulated in diverse types of cellular stresses and functions as either a pro-proliferation or pro-apoptosis factor depending on the specific microenvironment [9]. Some studies have shown that the ERS-induced TRB3 signaling activation increases cell apoptosis [10]; however, TRB3 may also participate in a negative feedback loop that regulates ATF4 and CHOP in ERS, protecting cells from apoptosis [11]. Our interest in TRB3 was initiated when we used Agilent Whole Rat Genome Oligo Microarrays to identify TRB3 and the ERS-related genes, GRP78, GRP94, and ATF4 as upregulated genes in hypoxic PASMCs [12]. What's more, the relationship between ERS and TRB3 has not been reported in hypoxic PASMCs. Emerging evidence shows that TRB3 has strikingly different roles in cancers, metabolic diseases and neurodegenerative diseases depending on the different cellular signaling pathways with which TRB3 interacts [13]. The MAPK signaling pathway was proved to play an important role in promoting the proliferation, migration and apoptosis resistance of PASMCs [14, 15]. The interaction between TRB3 and the MAPK signaling pathway has been explored in many cell types, however, the relationship between TRB3 and MAPK signaling pathway has not been defined in hypoxic PASMCs.

The purpose of the present study was to investigate the role and underlying mechanisms of TRB3 in hypoxic pulmonary vascular remodeling by overexpression or downregulation of TRB3, so as to find new targets for the treatment of hypoxic PH.

## Methods

### Ethics statement

All experiments were approved by the Huazhong University of Science and Technology Committee and the Tongji Medical College Ethics Committee, Tongji Hospital and were performed in compliance with the Guide for the Care and Use of Laboratory Animals of the National Institutes of Health.

### Cell culture and hypoxia treatment

Rat PASMCs were cultured using direct adherent culture methods. In brief, male Sprague–Dawley rats weighing 100 g to 120 g were obtained from the Laboratory Animal Services Center, Tongji Medical College and were intraperitoneally anesthetized using 7% chloraldurate. Rat lungs were harvested and placed in cold PBS. Then, the intralobar pulmonary arteries (PAs) were gently separated, and endothelial cells were removed using a cotton swab. Next, the PAs were cut into pieces, transferred to culture bottles, and spread separately on the bottle wall and the tissues were placed in a CO_2_ incubator at 37 °C. After 20 min, DMEM with 20% fetal bovine serum (FBS) was added to the culture bottle. Four to seven days later, the cells were identified by the method described in our previous study [5]. Rat PASMCs were exposed to 5% oxygen for hypoxia exposure in an airtight chamber (Billups-Rothberg) gassed with 5% CO_2_ and 90% N_2_.

### Lentivirus transduction

#### TRB3 silence

Short-hairpin RNAs targeted against rat TRB3 and the negative control sequence inserted into lentiviral vectors were obtained from Hanbio Biotechnology Co., Ltd. (Shanghai, China). The siRNA sequences targeting TRB3 were as follows: si-1, CTGCTACATCCCTGGTTGA; and si-2, GCACAGAGTACACCTGCAA. The negative control sequence (si-NC) was TTCTCCGAACGTGTCACGTAA.

#### TRB3 overexpression

The CDS of the rat TRB3 gene was cloned into the lentiviral vector by Hanbio Biotechnology Co., Ltd.

Cells (5 × 10^4^ cells) were seeded and reached 30–50% confluence the next day. Cells were transfected with the lentivirus according to the manufacturer's instructions. After 24 h, the medium containing the transfection mixture was removed, and fresh cell culture medium was added.

### Cell viability assay

Cells were seeded at a density of 3 × 10^3^ cells per well in a 96-well plate followed by preincubation with lentivirus for 72 h. Cell Counting Kit-8 (CCK-8) reagent was diluted and added to each well according to the manufacturer's guidelines (Dojindo Laboratories, Tokyo, Japan). After incubation for 2 h, the optical density (OD) values were read by an ELx800 Universal Microplate Reader (Bio-Tek Instruments, Inc., Winooski, VT, USA) at 450 nm.

### EdU staining

The Cell-Light™ EdU Apollo®643 In Vitro Imaging Kit from RiboBio (Guangzhou, China) was used to quantify cell proliferation ability. In brief, cells were pretreated with lentivirus for the CCK-8 assay. Then, 100 μl of diluted EdU A solution was added and incubated for 2 h in a CO_2_ incubator. Then, the cells were fixed with 4% paraformaldehyde and incubated with 100 μl of 1 × Apollo® solution for 30 min in the dark, and 100 μl of 1 × Hoechst 33342 was added to each well for cell nuclei staining. Red and blue fluorescence was obtained using a fluorescence microscope (Olympus, Japan).

### Detection of cell apoptosis

Flow cytometry: Annexin V and propidium iodide staining (KeyGEN BioTECH, KGA108) were used to detect cell apoptosis. Floating cells and adherent cells were collected and incubated with 5 μl of Annexin V-FITC and 5 μl of PI in the dark for 15 min. Apoptosis cells were detected by flow cytometry (BD Biosciences, San Jose, CA, USA), and then the percentages of the early apoptotic (Annexin V+ /PI −) and late apoptotic (Annexin V +/PI +) cells were analyzed.

Hoechst 33342 staining (Thermo Fisher Scientific Inc., #62249): Cells were washed twice and fixed with 4% paraformaldehyde. After washing twice with PBS, Hoechst 33342 solution was added, and cells were incubated for 10 min. After removal the Hoechst 33342 solution, cells were washed twice. Then the fluorescence was then examined using a fluorescence microscope (Olympus, Japan). The nuclei of healthy cells are generally spherical, and the DNA is evenly distributed. During apoptosis, the DNA becomes condensed, but this process does not occur during necrosis. Nuclear condensation can therefore be used to distinguish apoptotic cells from healthy cells or necrotic cells. Apoptotic cells were distinguished from the normal cells under fluorescence microscope by the forms of blue fluorescence enhancement or fragmentation.

### Cell migration assay

Cells (2 × 10^4^) were added to the upper chamber, and 600 μl of DMEM was added to the lower chamber in a 24-well Transwell system. After 24 h, serum-free medium was placed in the upper chamber, and medium containing 15% fetal bovine serum was added to the lower chamber for 12 h. The migrated cells were stained with 10% crystal violet and imaged using an optical microscope (Zeiss, Oberkochen, Germany), and quantitative analysis was performed to count the migrated cells in five randomly obtained fields.

### Western blot analysis

Lung tissues and PASMCs were lysed with RIPA lysis buffer. Lysates were centrifuged at 12,000 rpm at 4 °C for 15 min. Supernatants were collected, and the protein concentration was measured using a BCA kit. Equal amounts of protein were separated on sodium dodecyl sulfate polyacrylamide gels and transferred to polyvinylidene fluoride membranes. The following primary antibodies were used.

Polyclonal antibodies against PCNA, BAX, caspase 3, PARP, HRP-conjugated β-actin, and Survivin were purchased from Proteintech (USA); anti-Bcl2 antibody was obtained from Boster (China); anti-MMP9 antibody was obtained from Affinity (USA); anti-p38/p-p38 MAPK, anti-JNK/p-JNK, anti-ERK/p-ERK, and normal rabbit IgG antibodies were acquired from Cell Signaling Technology (USA); normal mouse IgG was obtained from Santa Cruz Biotechnology (USA). Western blots were visualized using the ChemiDocTM XRS+ imaging system.

### Immunoprecipitation

Each group of PASMCs was cultured in a 10 cm dish, and a small part of the cell lysates was used for western blotting. The remaining cell lysates were divided into two equal parts and incubated with primary antibody or normal control IgG. Then, protein A/G magnetic beads pretreated with immunoprecipitation lysis buffer were added and incubated. The immune complex was washed with RIPA lysis buffer, mixed with 5 × loading buffer, and then boiled for western blot analysis.

### Rat hypoxic PH model

Adult male Sprague–Dawley rats were purchased from the Laboratory Animal Center, Tongji Medical College. The rats were placed in a normobaric chamber (Oxycycler model A84XOV; BioSpherix, Lacona, NY), and the oxygen concentration was adjusted to 10% for 4 w for hypoxia treatment.

Adeno-associated virus type 1(AVV1) vectors encoding short-hairpin RNAs against rat TRB3 were constructed and purchased from Hanbio Biotechnology Co., Ltd. (Shanghai, China). In the prevention protocol, rats were airway injected with: AAV1-TRB3 or the negative control (AVV1-NC) before hypoxia treatment. Fifteen days after establishing the animal model, rats from the hypoxia group were airway injected with AAV1-TRB3 for the treatment group. The PH model rats were evaluated after exposure to hypoxia for 4 weeks. Hemodynamic studies and right ventricular hypertrophy were evaluated as previously reported [16]. The left lungs of the rats were fixed for histological analysis. The PAs of the right lungs were frozen in liquid nitrogen and transferred to a − 80 ℃ refrigerator for preservation.

### HE and Immunohistochemistry staining

Paraffin sections of the lung were stained with hematoxylin–eosin (HE), anti-α-smooth muscle actin (α-SMA) (1:100; Boster Biological Technology, Wuhan, China), anti-proliferating cell nuclear antigen (PCNA) (1:100; Proteintech Group), and anti-TRB3 (1:100; Proteintech Group).

### Measurement of medial wall thickness

Paraffin sections stained for α-SMA were imaged using light microscopy (Olympus, Tokyo, Japan), and the medial wall thickness was calculated using the following equation: medial wall thickness = [(external diameter − internal diameter)/external diameter].

### Clinical samples

Four pulmonary hypertension tissues and four normal pulmonary pressure lung tissues were obtained from the Department of Thoracic Surgery of Tongji Hospital. Informed consent was obtained from all subjects, and the study was approved by the Ethics Committee of Tongji Hospital, Tongji Medical College, Huazhong University of Science and Technology. The four PH patients were diagnosed with congenital heart disease, idiopathic pulmonary hypertension, chronic obstructive pulmonary disease and interstitial lung disease before the lung transplant. The four control lung tissues were from the edge of the mass and were excised from four lung adenocarcinoma patients.

### Statistical analysis

Statistical analyses were analyzed using GraphPad Prism (version 5.0). Student’s t-test was used for two-group comparisons. Comparisons between multiple groups were analyzed by one-way analysis of variance followed by Duncan’s test. P < 0.05 was considered statistically significant.

## Results

### TRB3 expression was upregulated by hypoxia and controlled by ERS

**Fig. 1:**
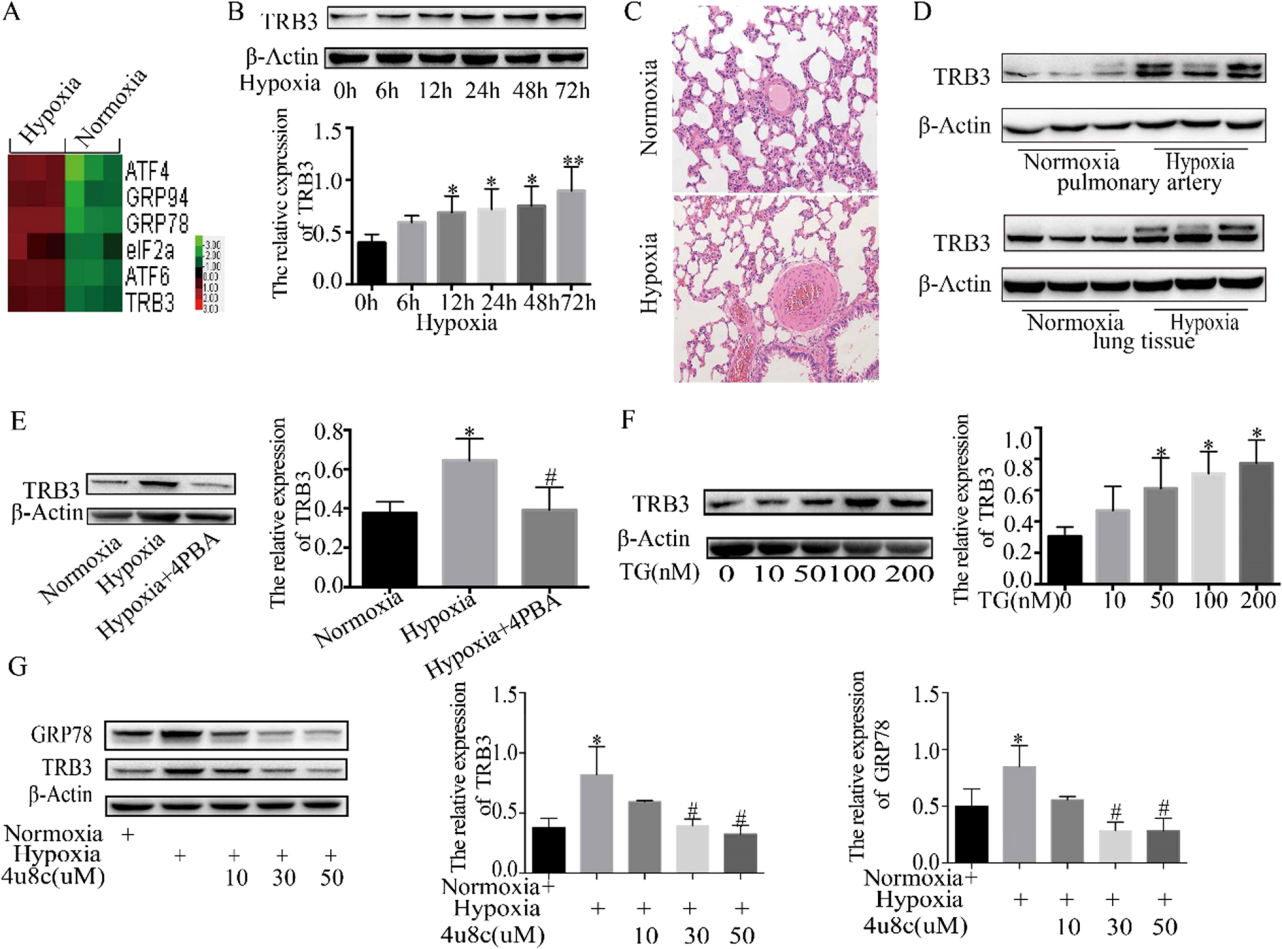
The expression of TRB3 was regulated by hypoxia and ERS. **A** A heat map of the hierarchical clustering of gene expression profiling of rat PASMCs for ERS from the Agilent Whole Rat Genome Oligo Microarrays. Each gene is depicted by one row, in which the red color denotes an increase in gene expression and the green color denotes a decrease in gene expression compared to the other group. Brighter colors represent higher gene expression levels. **B** TRB3 protein expression in PASMCs treated with hypoxia for different times. **C** The rats in the hypoxia group were exposed to 10% O_2_ for 8 h a day, and after 4 weeks, lung tissues were stained with hematoxylin–eosin (HE). **D** TRB3 protein expression in lung tissues and pulmonary arteries. **E**–**G** TRB3 protein expression in PASMCs treated with 4-PBA, TG, and 4μ8C. Data represent the mean ± SEM (n = 4). *P < 0.05 and **P < 0.01 compared to the group exposed to hypoxia for 0 h. ^#^P < 0.05 and ^##^P < 0.01 compared to PASMCs exposed to hypoxia for 48 h

A heat map of the hierarchical clustering of gene expression profiling from the Agilent Whole Rat Genome Oligo Microarrays revealed that the mRNA expression of TRB3 and the ERS-related genes, GRP78, GRP94, and ATF4, was upregulated in hypoxic PASMCs (Fig. 1A). TRB3 protein expression was further evaluated by western blot analysis. Exposure of rat PASMCs to different time points (0, 6, 12, 24, 48, and 72 h) of hypoxia resulted in upregulated TRB3 protein expression in a time-dependent manner (Fig. 1B). HE staining of lung tissues showed that hypoxia-induced pulmonary artery remodeling was successfully established (Fig. 1C) under our hypoxic treatment. Analysis of TRB3 expression in isolated pulmonary arteries (PAs) and lung tissues from hypoxic PH and normal lungs showed that TRB3 was upregulated in hypoxic PH models (Fig. 1D).

Our previous study identified that hypoxia activates ERS in PASMCs [5]. TRB3 is a stress sensor, and multiple studies have shown that the expression of TRB3 is affected by hypoxia and ERS [9]. We further explored the relationship between TRB3 and ERS. Upon stress, GRP78 is sequestered by misfolded proteins, leading to activation of the corresponding three unfolded protein response (UPR) branches to restore ER homeostasis, and it is considered a marker of ERS. We found that TRB3 expression in PASMCs was repressed by 4-PBA (Fig. 1E), an inhibitor of ERS, and upregulated by thapsigargin (TG) (Fig. 1F), an activator of ERS. Our previous study showed that the IRE1α/XBP1 pathway is activated by hypoxia in PASMCs to respond to the UPR. In the present study, 4μ8C, an inhibitor of the IRE1α/XBP1 pathway, was used to treat hypoxic PASMCs, demonstrating that 4μ8C repressed ERS and the protein expression of TRB3 in hypoxic PASMCs (Fig. 1G).

### TRB3 knockdown promoted apoptosis and attenuated the proliferation and migration of hypoxic PASMCs

**Fig. 2:**
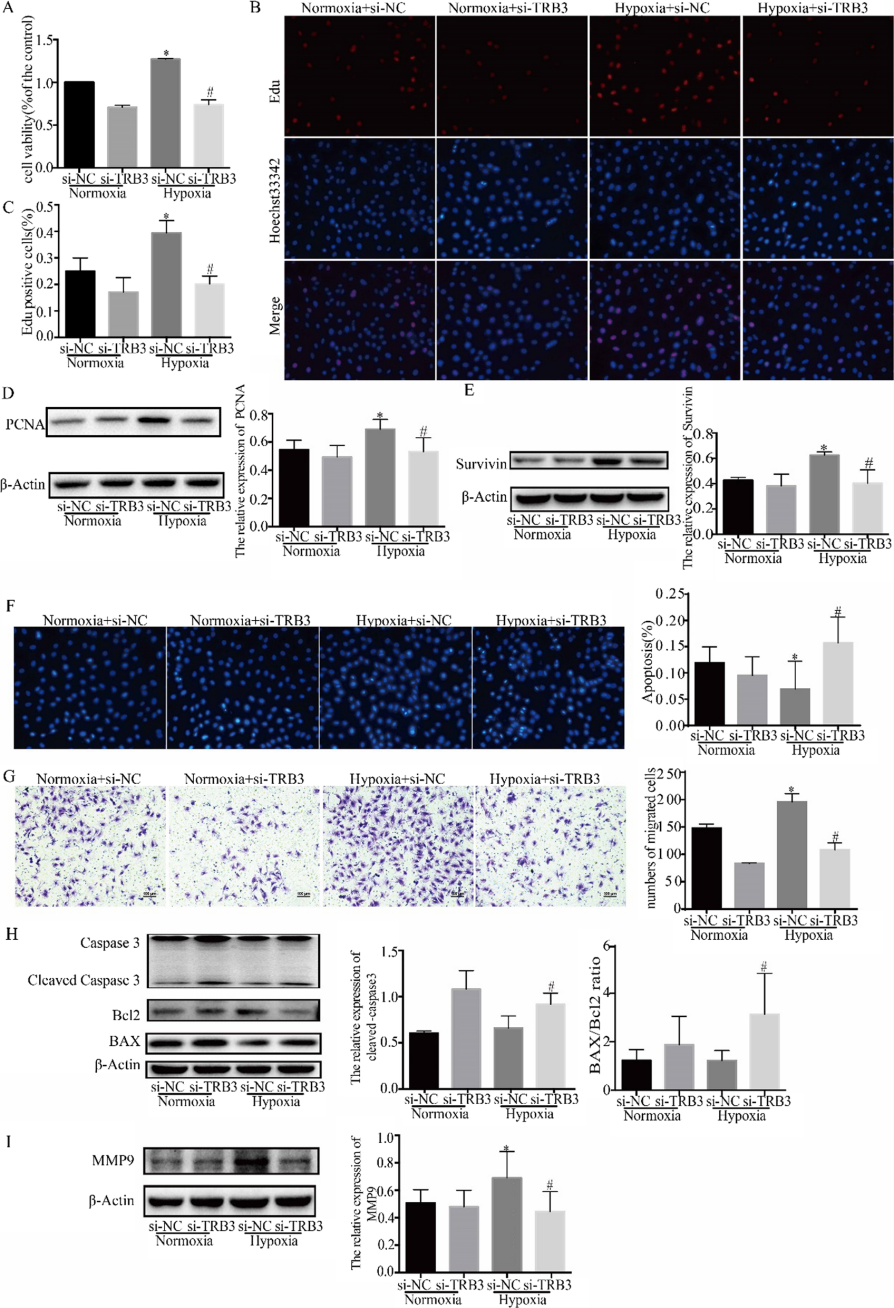
Effect of TRB3 knockdown on proliferation, migration, and apoptosis of hypoxic PASMCs. PASMCs were transfected with lentiviral vectors encoding short hairpin RNAs targeting against rat TRB3(si-TRB3) or negative control(si-NC) for further analysis. **A** CCK-8 assays were performed to test cell viability after TRB3 knockdown in hypoxic PASMCs. **B** The EdU proliferation assay was used to measure cell proliferation. All images are × 400 magnification. **C** EdU‐positive cells were analyzed. **D** and **E** The expression of PCNA and Survivin was examined by western blot, and the gray value was quantified by ImageJ software. **F** Cell apoptosis was evaluated by Hoechst 33342 staining, and the percentage of apoptosis was quantified. **G** Crystal violet staining of PASMCs is presented at × 200 magnification for the Transwell assay, and the migrated cells were counted manually. **H** Apoptosis-related protein expression was evaluated and quantification of the analysis is shown. **I** The expression of MMP9 was evaluated. Data represent the mean ± SEM (n = 4). *P < 0.05 and **P < 0.01 compared to normoxic PASMCs transfected with si-NC. ^#^P < 0.05 and ^##^P < 0.01 compared to hypoxic PASMCs transfected with si-TRB3. si-NC, lentiviral vectors encoding the negative control sequence; si-TRB3, lentiviral vectors encoding the short-hairpin RNAs against rat TRB3; PCNA, proliferating cell nuclear antigen; TRB3, Tribbles homolog 3; BAX, BCL2 associated X; Bcl2, B cell leukemia/lymphoma 2; MMP9, matrix metallopeptidase 9

The efficiency of the TRB3-silencing lentivirus was evaluated by measuring the mRNA and protein expression of TRB3 in PASMCs, and the best sequence was selected for further study. The CCK-8 test and EdU staining were performed to assess cell proliferation ability. CCK-8 tests showed that cell viability was enhanced in the hypoxia group, and TRB3 knockdown attenuated the viability of hypoxic PASMCs (Fig. 2A). The percentage of EdU-positive PASMCs was higher in the hypoxia group than in the normoxia group. TRB3 depletion induced a visible decrease in the percentage of EdU-positive nuclei (Fig. 2B and C). Moreover, the expression of the proliferation-related proteins, Survivin and PCNA, was upregulated in hypoxia, and TRB3 knockdown suppressed the protein expression of Survivin and PCNA in hypoxic PASMCs (Fig. 2D and E). Hoechst 33342 staining was used to identify apoptotic cells. As shown in Fig. 2F, the percentage of nuclear condensation cells was decreased in the hypoxia group compared to the normoxia group, and TRB3 knockdown induced a significant increase in the percentage of apoptotic cells. TRB3 depletion attenuated cell migration ability, which was evaluated by the Transwell assay (Fig. 2G). In addition, TRB3 knockdown upregulated the expression of apoptosis-related proteins, including cleaved-caspase3, and the BAX/Bcl2 ratio was increased after TRB3 knockdown (Fig. 2H). The expression of the migration-related protein, MMP9, was upregulated in hypoxia, and TRB3 knockdown decreased the protein expression of MMP9 (Fig. 2I). The results of Fig. 2 demonstrated that the increase of cell proliferation and migration that occurred under hypoxic conditions were attenuated in the cells transfected with si-TRB3, and apoptotic cells were significantly increased.

### TRB3 overexpression enhanced cell proliferation and migration but inhibited cell apoptosis

**Fig. 3:**
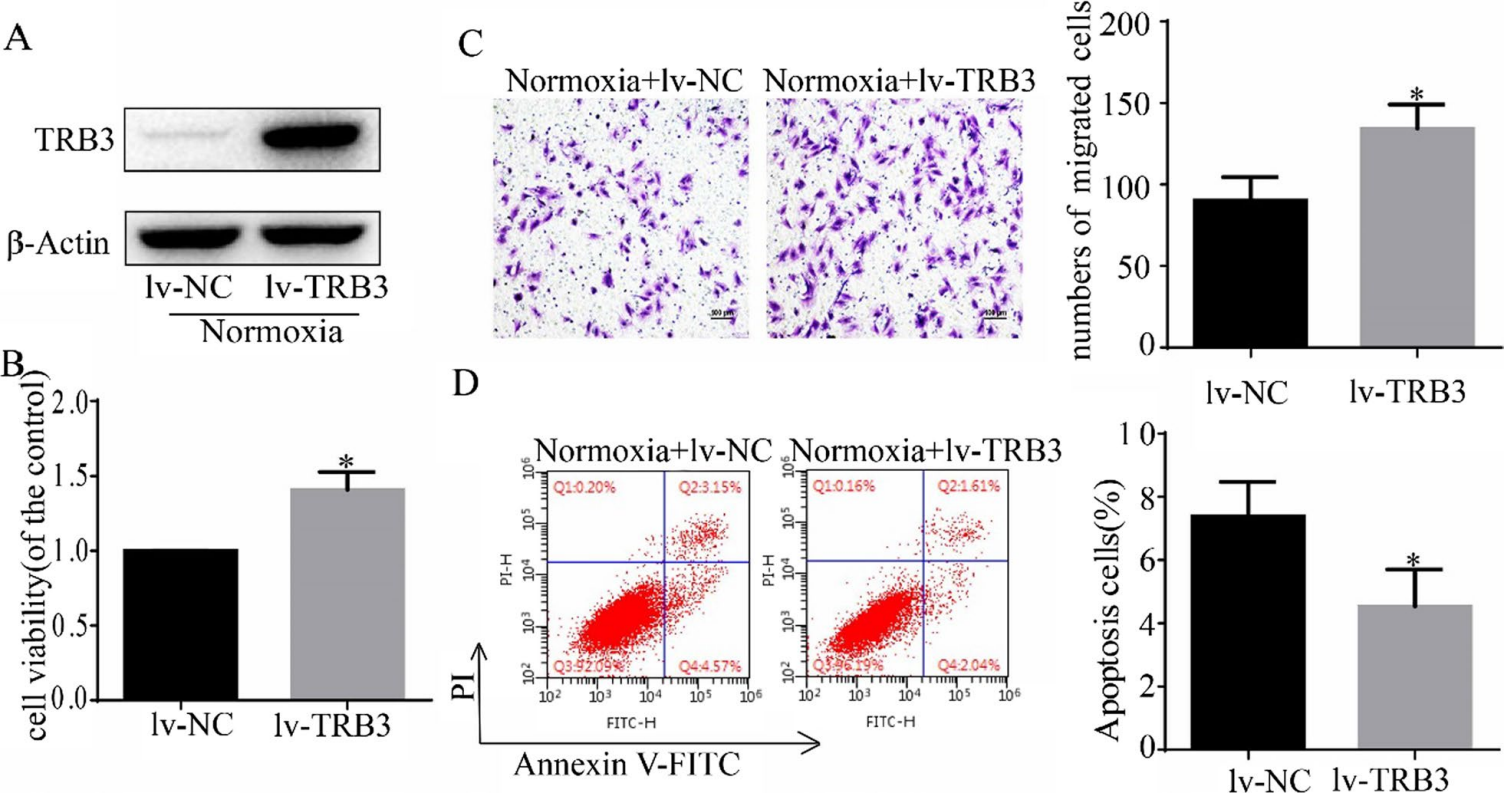
TRB3 overexpression in PASMCs increased cell proliferation and migration but impeded cell apoptosis. **A** TRB3 overexpression was achieved by lentiviral transfection, and the efficiency was determined by Western blot analysis. **B** The CCK8 test was used to determine cell proliferation. **C** Crystal violet staining is presented at × 200 magnification for the Transwell assay, and the migrated cells were counted. **D** Cell apoptosis induced by TRB3 overexpression was detected using flow cytometry and the percentage of early apoptotic (Annexin V+/PI−) and late apoptotic (Annexin V+/PI+) cells was determined. Data represent the mean ± SEM (n = 4). *P < 0.05 compared to the normoxic PASMCs transfected with lv-NC. lv-TRB3, PASMCs transfected with a lentiviral vector containing TRB3 CDS; lv-NC (negative control), PASMCs transfected with an empty lentiviral vector

TRB3 was overexpressed in normoxic PASMCs, and the transfection efficiency was determined by Western blot analysis (Fig. 3A). Cell viability, cell migration, and cell apoptosis were also assessed. The CCK-8 assay (Fig. 3B) and Transwell assay (Fig. 3C) showed that TRB3 overexpression increased PASMC proliferation and migratory ability. Flow cytometry was adopted to examine apoptotic rate which indicated that TRB3 overexpression inhibited cell apoptosis ability by analyzing the percentage of early apoptotic (Annexin V+/PI−) and late apoptotic (Annexin V +/PI +) cells (Fig. 3D).

### TRB3 knockdown inhibited but TRB3 overexpression augmented the activation of the MAPK signaling pathway

**Fig. 4:**
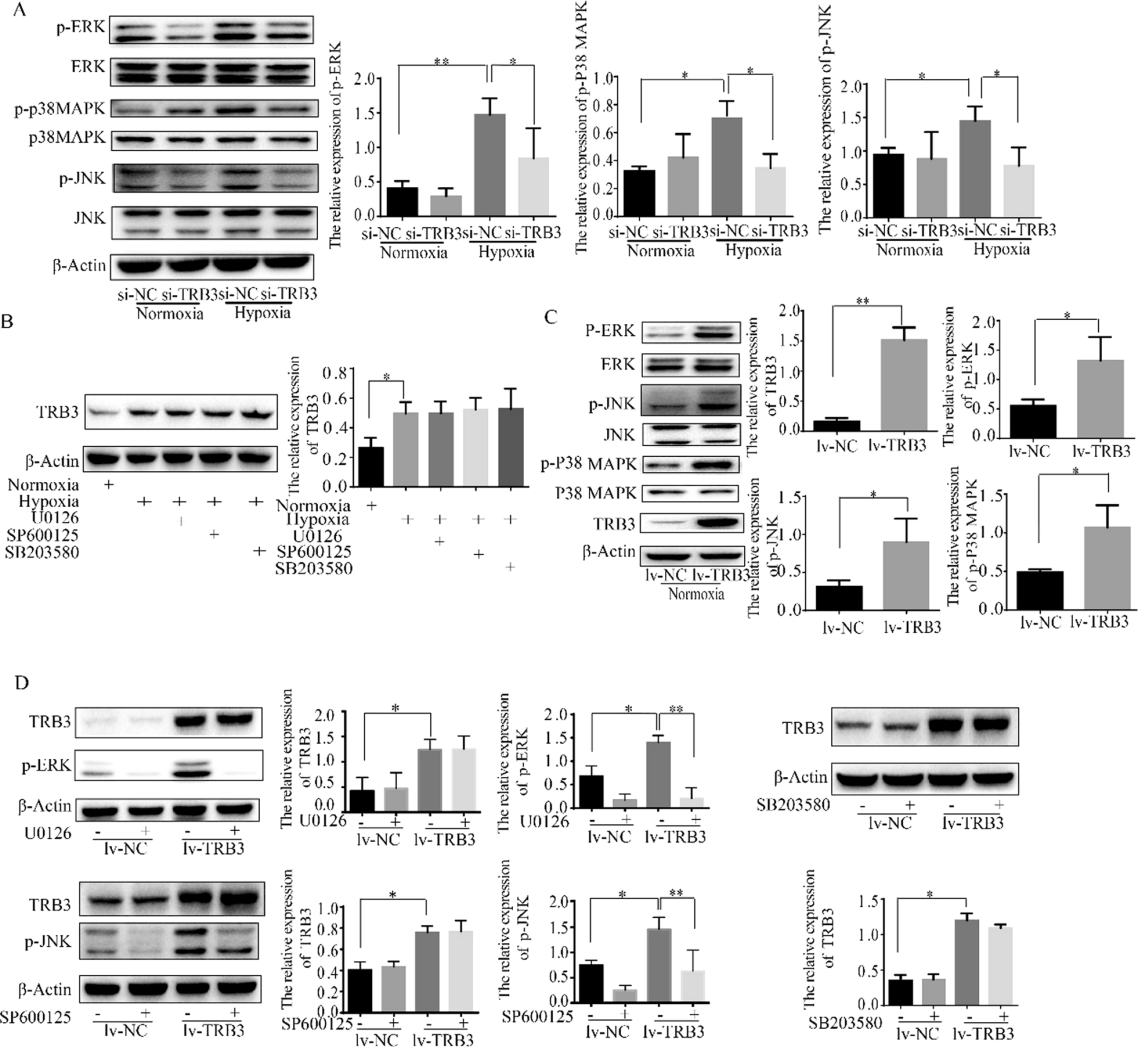
The connection of TRB3 and MAPK signaling in PASMCs was explored by western blot analysis. **A** and **C** Western blot analysis of the phosphorylation of ERK, JNK, and p38 MAPK in PASMCs after TRB3 knockdown and overexpression. **B** Effect of ERK, JNK, and p38 MAPK inhibitors on TRB3 in hypoxic PASMCs by Western blot analysis. **D** Effect of ERK, JNK, and p38 MAPK inhibitors on TRB3 in PASMCs transfected with a lentiviral vector expressing TRB3 (lv-TRB3) by Western blot analysis. Data represent the mean ± SEM (n = 4). *P < 0.05 and **P < 0.01. ERK, extracellular signal‐regulated kinase; JNK, c‐Jun N‐terminal kinase; p38 MAPK, p38 mitogen-activated protein kinase (MAPK)

The MAPK signaling pathway mediates the process of signal transmission and various physiological processes such as cell proliferation, transformation, and apoptosis [17]. It has been demonstrated that TRB3 controls both the extent and specificity of MAPK kinase activation of MAPK [18]. The regulatory effects of TRB3 on MAPK signaling have been demonstrated to regulate cell growth in cardiac fibrosis [19] and many types of cancers [20, 21]. The connection of TRB3 and MAPK signaling in PASMCs was explored in the present study. In hypoxic PASMCs, TRB3 knockdown inhibited the phosphorylation of ERK, JNK and p38 MAPK (Fig. 4A). However, TRB3 protein expression in hypoxic PASMCs was not affected by the ERK inhibitor (U0126), JNK inhibitor (SP600125), or p38 MAPK inhibitor (SB203580) (Fig. 4B). We further overexpressed TRB3 in normoxic PASMCs and found that TRB3 overexpression increased the protein expression of p-ERK, p-JNK, and p-p38 MAPK (Fig. 4C). Whereas, the ERK inhibitor (U0126), JNK inhibitor (SP600125), and p38 MAPK inhibitor (SB203580) did not affect the expression of TRB3 in TRB3-overexpressing PASMCs (Fig. 4D). The experimental results indicated that TRB3 is an upstream regulator of the ERK, JNK, and p38 MAPK pathways.

### TRB3 regulated PASMC biological behavior by affecting the MAPK signaling pathway

**Fig. 5:**
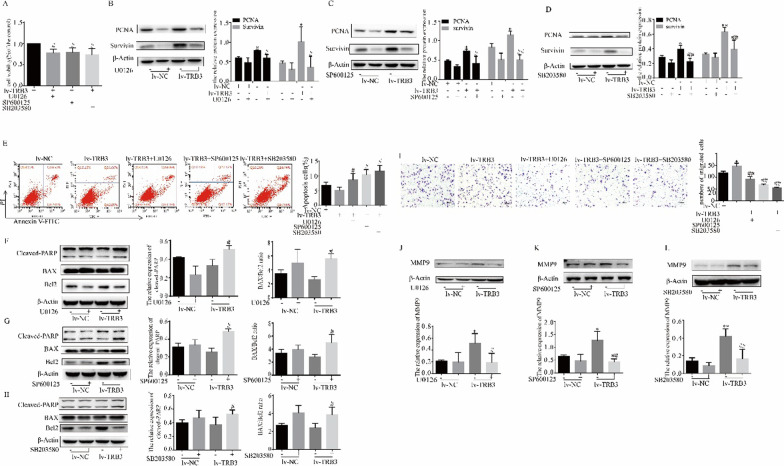
Inhibitors of ERK, JNK, and p38 MAPK reversed the effect of TRB3 overexpression on PASMC biological behavior. **A** The CCK-8 assay was used to evaluated the proliferation of TRB3-overexpressing cells after treating with U0126, SP600125, and SB203580. **B**–**D** Western blot analysis of PCNA expression in TRB3 overexpressing cells incubated with U0126, SP600125, or SB203580 for 12 h. **E** Cell apoptosis induced by U0126, SP600125 and SB203580 in TRB3-overexpressing cells was evaluated using flow cytometry, and the percentage of early apoptotic (Annexin V+/PI−) and late apoptotic (Annexin V+ /PI+) cells was analyzed. **F**–**H** Western blot analysis of the protein expression of PARP, BAX, and Bcl2 in TRB3-overexpressing cells incubated with U0126, SP600125, and SB203580 for 12 h. **I** Crystal violet staining is presented at × 200 magnification for the Transwell assay and the migrated cells were counted and analyzed. **J**–**L** Western blot analysis of MMP9 expression in TRB3-overexpressing cells incubated with U0126, SP600125, and SB203580 for 12 h. Data represent the mean ± SEM (n = 4). *P < 0.05 compared to normoxic PASMCs transfected with lv-NC. ^#^P < 0.05 and ^##^P < 0.01 compared to PASMCs transfected with lv-TRB3. U0126, an ERK signaling inhibitor; SP600125, an JNK signaling inhibitor; SB203580, an p38 MAPK signaling inhibitor

Next, we investigated whether the activation of MAPK signaling mediated TRB3 induction of PASMC biological behavior. Inhibitors of the ERK pathway, JNK pathway, and p38 MAPK pathway abolished the effect of TRB3 overexpression on the regulation of PASMC proliferation (Fig. 5A), apoptosis (Fig. 5E), and migration (Fig. 5I). Inhibitors of the ERK, JNK, and p38 MAPK pathways reversed the proliferation- (Fig. 5B–D), apoptosis- (Fig. 5F–H), and migration- (Fig. 5J–L) related protein expression in TRB3 overexpressing PASMCs. The results indicated that the MAPK signaling pathway mediated the changes of the biological behavior in TRB3-overexpressing cells.

### TRB3 interacted with ERK, JNK, and p38 MAPK

**Fig. 6:**
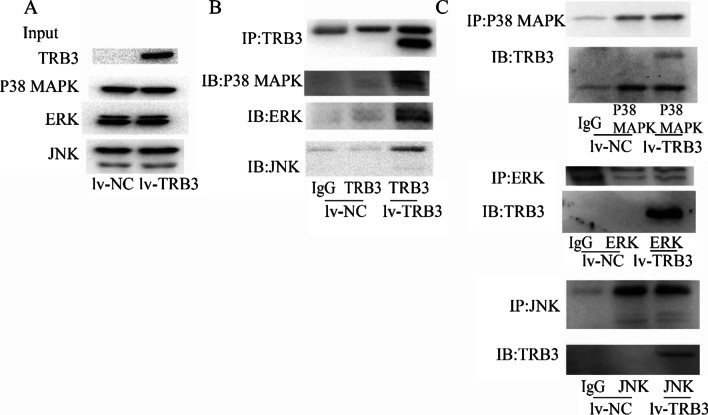
Co-immunoprecipitation (IP) analysis was performed to examine the association between TRB3 and the MAPK signaling pathway. **A** The protein expression of TRB3, ERK, JNK and p38 MAPK in whole cell extracts was evaluated in PASMCs transfected with a negative control vector(lv-NC) or lentiviral vector containing TRB3 CDS (lv-TRB3). **B** Total cell extracts were immunoprecipitated with an anti‐TRB3 antibody followed by blotting with anti‐ERK, anti‐JNK, and anti-p38 MAPK antibodies. **C** Whole‐cell extracts were separately immunoprecipitated with anti‐ERK, anti‐JNK, and anti-p38 MAPK antibodies followed by blotting with anti‐TRB3

The relationship of TRB3 with ERK, JNK, and p38 MAPK was further studied by coimmunoprecipitation assay. Figure 6A shows that the same amount of ERK, JNK, and p38 MAPK was used for immunoprecipitation assay for TRB3 overexpressed cells and the control cells. TRB3 was demonstrated to coimmunoprecipitate with ERK, JNK, and p38 MAPK (Fig. 6B) and vice versa (Fig. 6C). In comparison with the cells transfected with the negative control lentivirus, TRB3 overexpression increased the amount of ERK, JNK, and p38 MAPK protein coprecipitating with TRB3.

### Intratracheal delivery of AAV1-TRB3 shRNA reversed established hypoxic PH and prevented the pathological process of hypoxia-induced PH

**Fig. 7:**
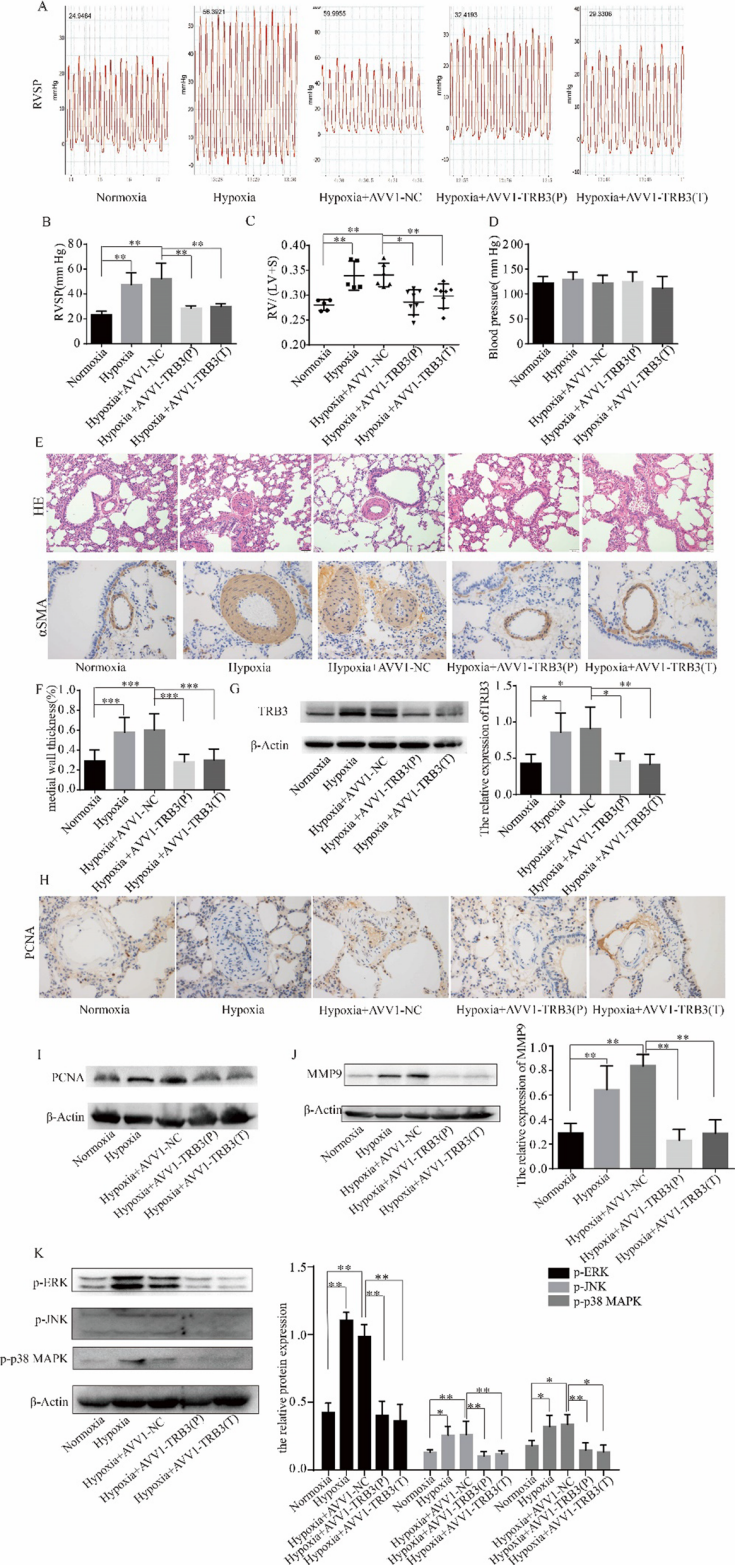
TRB3 knockdown reversed and prevented the progression of hypoxia -induced PH. **A** The right ventricular systolic pressure(RVSP) was measured by a 1.2-Fr pressure catheter (GuGeShengWu, Wuhan, China) inserted via the right jugular vein and positioned in the right ventricle. **B** The values of RVSP were obtained using the ADInstruments Powerlab data acquisition system. **C** Right ventricular hypertrophy was evaluated by the ratio of the RV/(LV + S). The right ventricle (RV) was peeled off from the left ventricle (LV) and the septum (S), and the dry weight of the two components was measured. **D** Systemic blood pressure was measured by a 1.2-Fr pressure catheter inserted via the left carotid artery. **E** Lung tissue sections were stained for hematoxylin–eosin (HE) (magnification, × 200) and α-SMA (magnification, × 400). **F** Pulmonary vascular remodeling was evaluated by the percentage of medial wall thickness of distal pulmonary vessels, which was expressed using the following equation: [(external diameter − internal diameter)/external diameter]. **G** The whole pulmonary artery of each rat from different groups was extracted, and TRB3 protein expression in each group was measured by western blot analysis. **H** and **I** PCNA protein expression in pulmonary arteries was examined by immunohistochemical staining and western blot analysis. **J** MMP9 protein expression in pulmonary arteries was examined by western blot analysis. **K** The phosphorylated forms of ERK, JNK, and p38 MAPK in pulmonary arteries in each group were evaluated by western blot analysis. Data represent the mean ± SEM (n = 4). *P < 0.05 and **P < 0.01

As TRB3 was upregulated in hypoxic PASMCs and mediated the proliferation-enhanced and apoptosis-resistant phenotype of hypoxic PASMCs in vitro, we next evaluated the effect of TRB3 silencing on pulmonary hemodynamics and vascular remodeling in a hypoxic PH model. The results of hemodynamic changes showed that TRB3 silencing had therapeutic and preventative efficacy. TRB3 silencing reduced right ventricular systolic pressure (RVSP) (Fig. 7A, B) and relieved ventricular hypertrophy as indicated by the RV/(LV + S) ratio (Fig. 7C), and silencing TRB3 did not affect systemic blood pressure (Fig. 7D). HE staining showed morphometric analysis of distal pulmonary arteries. Hypertrophy of small pulmonary arteries was observed in vessels of the PH rat model and was attenuated in AVV1-TRB3-treated rats. This finding was confirmed by demonstrating a decrease in the presence of α-SMA—positive cells in the medial layer of vessels from AVV1-TRB3-treated PH rats in comparison with the other groups. The ratio of wall thickness to the radius of vessels was analyzed, and the results showed that TRB3 silencing limited and reversed pulmonary artery remodeling in the prevention and treatment groups (Fig. 7E, F). We examined TRB3 protein expression in PAs at the end of hypoxia treatment. The results of immunoblot analysis confirmed that TRB3 protein expression was increased in pulmonary vessels from rats with hypoxia-induced PH, and airway injection of AAV1-TRB3 shRNA was sufficient to decrease PAs TRB3 expression in the prevention and treatment protocols (Fig. 7G).

We next confirmed the expression of the proliferation-related protein, PCNA, by immunohistochemical staining and western blot analysis. TRB3 silencing decreased the percentage of PCNA-positive PASMCs (Fig. 7H), and the protein expression of PCNA in PAs was downregulated by TRB3 silencing (Fig. 7I). Consistent with the decrease in PASMC proliferation, treatment with AAV1-TRB3 shRNA in hypoxic PH rats downregulated the expression of MMP9 (Fig. 7J) and decreased the phosphorylation and activation of ERK, JNK, and p38 MAPK (Fig. 7K) compared to AVV1-NC -treated PH rats.

### TRB3 expression was upregulated in PH patients

**Fig. 8:**
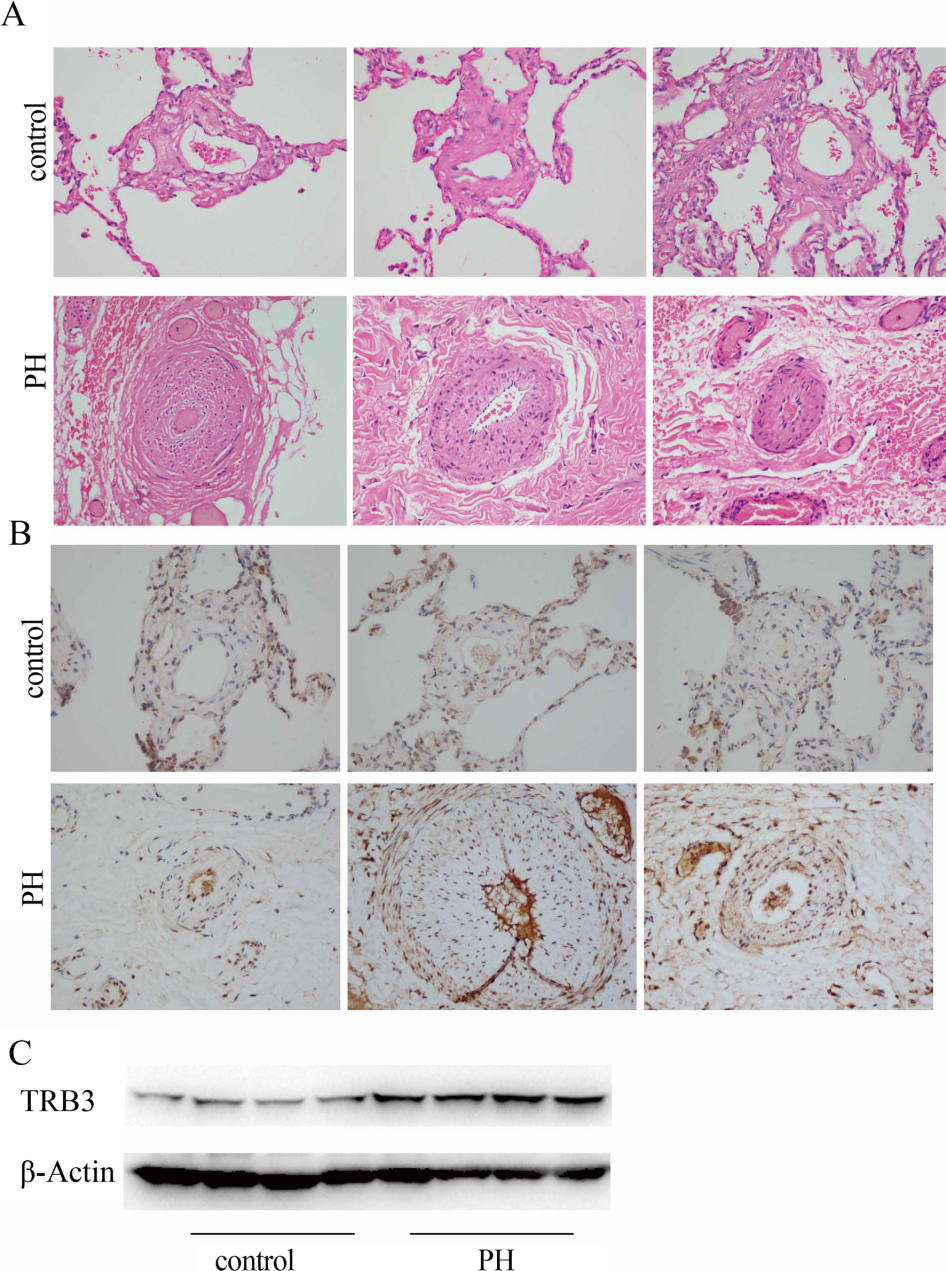
TRB3 protein expression was expressed at a higher level in PH patients. **A** Lung sections stained for hematoxylin–eosin (HE) (magnification, × 400). **B** TRB3 expression in pulmonary arteries in lung sections of the two groups stained for TRB3 (magnification, × 400). **C** TRB3 protein expression in pulmonary tissues of PH patients and controls was evaluated by western blot analysis

We collected human lung tissues from patients receiving lung transplants who had pulmonary hypertension and noncancerous lung tissues from lung cancer patients who did not have PH. Hypertrophy of pulmonary arteries was observed in the vessels of PH patients, as shown in Fig. 8A. The results of immunohistochemical staining and western blot analysis showed that TRB3 protein expression was upregulated in the pulmonary arteries (Fig. 8B) and lung tissues (Fig. 8C) of four PH patients compared to four patients with normal pulmonary pressure.

## Discussion

The present study provided the first evidence that TRB3 protein expression levels are upregulated in cultured hypoxic rat PASMCs and lung samples from a rat hypoxic PH model and PH patients. A series of cytological and molecular experiments were performed to clarify the underlying role and mechanisms of TRB3 in hypoxic PASMCs. TRB3 directly binds ERK, JNK, and p38 MAPK and then activates MAPK signaling, which is associated with enhanced proliferation, migration, and apoptosis resistance of PASMCs. Furthermore, AAV1-TRB3 shRNA was used to knockdown the expression of TRB3 in pulmonary arteries by previously published methods [22, 23], and TRB3 knockdown reversed and prevented hypoxia-induced pulmonary hypertension, right ventricular hypertrophy and pulmonary arterial remodeling in the hypoxic PH model. These findings indicated that TRB3 is a potential biomarker and promising target for PH therapy.

Multiple studies have demonstrated that TRB3 mediates various intracellular signaling transmissions by interacting with transcription factors or proteins to function as a scaffold protein. TRB3 has the tumor-promoting effects of insulin/IGF in hepatocellular cancer cells [24]. However, there is also evidence that TRB3 is a tumor suppressor that contributes to the antitumor effect of various antitumor drugs in hepatocellular carcinoma [25]. TRB3 plays a pro-death or pro-survival role and may be cell type- and stimulus-specific. To date, few studies have reported the role of TRB3 in idiopathic pulmonary artery hypertension (IPH) by regulation the BMP pathway [26, 27]; however, the role of TRB3 in hypoxic PH has not been elucidated.

TRB3 is recognized as an endogenous stress sensor and performs either pro-survival or pro-apoptotic functions in response to a range of environmental stimuli, such as ER stress [7], metabolism [28], and hypoxia [8], in a context-dependent manner, but the mechanisms are poorly understood. Here, we showed that the expression of TRB3 was increased in hypoxic PASMCs in a time-dependent manner and was regulated by the level of ERS in PASMCs. TG activated ERS and upregulated the expression of TRB3, and 4-PBA inhibited ERS and downregulated the expression TRB3. Attenuating ERS with 4-PBA was proved as a novel therapeutic strategy in pulmonary hypertension [6], TRB3 maybe a potent target of ERS inhibitors in decreasing pulmonary artery remodeling. Whereas, it needs more experimental evidence to support.

Excessive proliferation, migration, and apoptotic resistance of PASMCs are hallmarks of hypoxic PASMCs in hypoxic pulmonary arterial remodeling and pulmonary hypertension. This study provided insight into PASMC biological behavior in hypoxic pulmonary vascular remodeling. TRB3 knockdown suppressed the proliferation and migration of hypoxic PASMCs but promoted apoptosis. In contrast, TRB3 overexpression under normoxic conditions enhanced the proliferation and migration abilities but impaired the apoptosis ability of PASMCs.

The molecular mechanisms underlying the TRB3-mediated regulation of biological behavior in PASMCs have not yet been thoroughly addressed. The three MAPK families, namely, extracellular signal-regulated kinase (ERK), Jun kinase (JNK) and p38 MAPK, have been characterized to regulate proliferation, differentiation, transformation, and apoptosis. The interaction between TRB3 and the MAPK signaling pathway has been explored in breast cancer, demonstrated that TRB3 is a master regulator of Notch through the ERK and TGFβ pathways [20]. In renal cell carcinoma and pulmonary interstitial fibrosis, TRB3 enhances cell viability and invasiveness by targeting the ERK, JNK, and p38 MAPK signaling pathways [21, 29]. The MAPK signaling pathway has been demonstrated to contribute to hypoxic pulmonary vascular remodeling by stimulating cell proliferation and migration and reducing apoptosis in PASMCs [14, 15, 30, 31]. The present study showed that in response to TRB3 knockdown or overexpression in PASMCs, MAPK signaling activation was repressed or activated because the phosphorylation of ERK, p38 MAPK and JNK was reduced in TRB3-depleted cells but increased in TRB3-overexpressing cells. However, TRB3 protein expression in hypoxic PASMCs and TRB3 overexpressing cells was not affected by inhibitors of the MAPK signaling pathway. Thus, the regulatory effects of TRB3 on ERK, JNK, and p38 MAPK indicated that TRB3 regulates the MAPK signaling pathway as an upstream regulator.

The present study also demonstrated that the activation of the MAPK signaling pathway accounted for TRB3-regulated PASMC biological behavior by evaluating PASMC proliferation, migration, and apoptosis. U0126, SP600125, and SB203580 were used to inhibit ERK, JNK, and p38 MAPK activation, respectively. The effects of TRB3 overexpression on PASMC proliferation, migration, and apoptosis were reversed by treatment with U0126, SP600125, or SB203580. The upregulation of PCNA, Survivin and MMP9 as well as the inhibition of cleaved-PARP and BAX/Bcl2 in TRB3-overexpressing cells were also abolished by the inhibitors of the three MAPK pathways.

TRB family members lack detectable kinase activity because they have a truncated kinase domain, hence they are called pseudokinases. However, TRB family members may bind to other proteins and modulate their function. TRB3 has been reported to bind to Akt and block the activation of Akt [25, 32] as well as bind to SAMD3 and promote the activation of SMAD3 [33]. TRB3 has also been demonstrated to interact with p62; interruption of the TRB3/p62 interaction produces potent antitumor efficacies, and a peptide targeting the interaction inhibits the interaction of TRB3/p62, resulting in significant antitumor effects [24]. The interaction of TRB3 with ERK, JNK, and p38 MAPK was investigated by coimmunoprecipitation assays in the present study. An enhanced amount of the direct binding protein of TRB3 with ERK, JNK and p38 MAPK in TRB3 overexpressing PASMCs with activation the MAPK signaling pathway was observed. Interrupting the TRB3–MAPK interaction with peptides may be a promising strategy for targeting hypoxic PH.

## Conclusions

TRB3 directly binds ERK, JNK, and p38 MAPK and then activates MAPK signaling, which plays a critical role in in hypoxia induced pulmonary vascular remodeling. TRB3 may be a potential therapeutic target against hypoxic PH.

## Data Availability

Any data generated and/or analysed during the current study are available from the corresponding author on reasonable request.

## Abbreviations

AVV1: Adeno-associated virus type 1
BAX: BCL2 associated X
Bcl2: B cell leukemia/lymphoma 2
CCK-8: Cell Counting Kit-8
ER: Endoplasmic reticulum
ERK: Extracellular signal‐regulated kinase
ERS: Endoplasmic reticulum stress
FBS: Fetal bovine serum
JNK: C‐Jun N‐terminal kinase
LV: Left ventricle
MMP9: Matrix metallopeptidase 9
OD: Optical density
PAs: Pulmonary arteries
PASMCs: Pulmonary artery smooth muscle cells
PCNA: Proliferating cell nuclear antigen
PH: Pulmonary hypertension
RV: Right ventricle
RVSP: Right ventricular systolic pressure
TG: Thapsigargin
TRB3: Tribbles homolog 3
UPR: Unfolded protein response
